# A controlled comparison of thickness, volume and surface areas from multiple cortical parcellation packages

**DOI:** 10.1186/s12859-019-2609-8

**Published:** 2019-01-28

**Authors:** Shadia S. Mikhael, Cyril Pernet

**Affiliations:** University of Edinburgh, Centre for Clinical Brain Sciences (CCBS), The Chancellor’s Building, 49 Little France Crescent, Edinburgh, EH16 4SB UK

**Keywords:** Cortical parcellation, Grey matter, Thickness, Volume, Surface area, Superior frontal gyrus, Supramarginal gyrus, Cingulate gyrus, Brain, Atlas

## Abstract

**Background:**

Cortical parcellation is an essential neuroimaging tool for identifying and characterizing morphometric and connectivity brain changes occurring with age and disease. A variety of software packages have been developed for parcellating the brain’s cortical surface into a variable number of regions but interpackage differences can undermine reproducibility. Using a ground truth dataset (*Edinburgh_NIH10)*, we investigated such differences for grey matter thickness (GM_th_), grey matter volume (GM_vol_) and white matter surface area (WM_sa_) for the superior frontal gyrus (SFG), supramarginal gyrus (SMG), and cingulate gyrus (CG) from 4 parcellation protocols as implemented in the FreeSurfer, BrainSuite, and BrainGyrusMapping (BGM) software packages.

**Results:**

Corresponding gyral definitions and morphometry approaches were not identical across the packages. As expected, there were differences in the bordering landmarks of each gyrus as well as in the manner in which variability was addressed. Rostral and caudal SFG and SMG boundaries differed, and in the event of a double CG occurrence, its upper fold was not always addressed. This led to a knock-on effect that was visible at the neighbouring gyri (e.g., knock-on effect at the SFG following CG definition) as well as gyral morphometric measurements of the affected gyri. Statistical analysis showed that the most consistent approaches were FreeSurfer’s Desikan-Killiany-Tourville (DKT) protocol for GM_th_ and BrainGyrusMapping for GM_vol_. Package consistency varied for WM_sa_, depending on the region of interest.

**Conclusions:**

Given the significance and implications that a parcellation protocol will have on the classification, and sometimes treatment, of subjects, it is essential to select the protocol which accurately represents their regions of interest and corresponding morphometrics, while embracing cortical variability.

**Electronic supplementary material:**

The online version of this article (10.1186/s12859-019-2609-8) contains supplementary material, which is available to authorized users.

## Background

Various magnetic resonance imaging (MRI) tools have been developed to characterise the changes that the human brain undergoes over the course of a lifetime. One way to characterize such changes is through surface-based modelling packages. Following the initial phase of pre-processing, the packages divide the brain into layers and parcels using a range of algorithms and atlases. Parcel morphometry is then interpreted through several metrics such as cortical thickness, or grey matter thickness (GM_th_ [1]), grey matter volume (GM_vol_ [2, 3]), white matter surface area (WM_sa_, [1]), sulcal length and depth [4], gyrification index [5, 6], and fractal dimensionality [7].

Morphometric analysis software tools are powerful techniques with multiple applications. Given their ability to examine critical cortical regions, they have proven essential for the identification of maturational changes (e.g. [8–10] and biomarkers of disease (e.g., application in multiple sclerosis [11]; autism spectrum disorder [12]; schizophrenia [13]; Alzheimer’s disease [14], amnestic and non-amnestic mild cognitive impairment [15] to only name a few). From a computational perspective, these tools show good repeatability (although OS variations can be an issue due to underlying libraries, see e.g., [16]) and reliability of measurements for the same individuals (e.g., [17]). From an anatomical perspective, some morphometric measurements have been validated against post-mortem analyses (for instance, Cardinale et al., [18] showed a good agreement between FreeSurfer cortical thickness estimations and histological measurements), whilst parcellation per se is typically assessed visually by experts, in comparison or not to manually prepared data (e.g., [19]). In our previous work, we investigated critical differences between popular brain image analysis tools with focus on their cortical parcellation protocols [20]. We identified a lack of details in terms of the reference populations used, inconsistencies in gyral border definitions, and uncertainties with variability considerations. We concluded with an emphasis on the need for such details due to the direct influences that the derived parcels would have on any consequent analysis. Here we present a controlled comparison between FreeSurfer, BrainSuite and BrainGyrusMapping to quantify how differences in algorithms and protocols led to differences in parcel metrics, in comparison to ground truth data [21].

## Methods

### Subjects

Publicly available MRI data from 10 healthy right-handed non-smokers (Table 1 - mean age 59.8) were used [22].

**Table 1:** Demographics of the 10 healthy subjects from the NIH-funded study

Subject ID	1	2	3	4	5	6	7	8	9	10
Age	57	56	63	64	64	57	59	61	62	55
Gender	M	M	F	F	M	F	F	F	M	M

5 male and 5 female right-handed subjects of mean age 59.8 were investigated

The subject data, including their T1 and T2-weighted volumes, are publically available in the Edinburgh DataShare repository [22] organized in Brain Imaging Data Structure (BIDS [23]).

### Data acquisition

All subjects were scanned at the Brain Research Imaging Centre, Edinburgh (UK) in a 1.5 T scanner (General Electric, Milwaukee, WI, USA). A coronal high resolution 3D T1-weighted (FSGE, 1*1.3*1 mm voxel size, TE 4.01 ms TR 9.8 ms flip angle 8°), an axial T2-weighted (SE, 1*1*2 mm voxel size, TE 104.9 ms TR 1320 ms flip angle 8°), and a T2 FLAIR volume were acquired for each subject, and reviewed by a consultant radiologist ensuring their good health. Additional details can be found in [21].

### Materials

We chose 3 existing software packages to analyse the raw T1w data of each of the 10 subjects: FreeSurfer [24–26], BrainSuite [3], and BrainGyrusMapping [2]. A Linux version of FreeSurfer version 6.0 (freesurfer-Linux-centos6_x86_64-stable-pub-v6.0.0-2beb96c) was downloaded onto the department’s server and run using the default *recon-all* command, which allowed us to compare their older Desikan-Killiany protocol [27] to its updated version, the Desikan-Killiany-Tourville protocol [19]. BrainSuite version 13a (build#1744, built with Qt 4.8.4 on Sept 112,013) was installed and run on a Windows 7, 64-bit operating system with 16G RAM, using the BrainSuite GUI. We used the default Cortical Surface Extraction Sequence, while refining the sulcal curves for accuracy. A BrainGyrusMapping (BGM, v 11.0.3888 beta = v 1.0) command-line tool was provided by Canon Medical Research Europe^1^ and installed on the same Windows 7 system. This latter tool is a multi-atlas segmentation tool, originally built and validated using the data from the Medical Image Computing and Computer Assisted Intervention (MICCAI) 2012 challenge on multi-atlas labelling [2]. We selected the maximum number of atlases, 28, to be used by this tool rather than the default number, 7. All tools aside from BGM are freely available to the public. BGM’s parcellation protocol is freely available as well [28]. We additionally ran each tool 3 times on the same platform to assess its repeatability.

The results from these tools were compared to those of our morphometrics tool, Masks2Metrics [29, 30], which we ran on the same data with corresponding consistent ground truth. Briefly, the T1 and T2 images were combined to enhance grey-white matter borders and parcels drawn manually using a detailed protocol which accounted for all known anatomical variability (see [21] for details and validation). Using this ground truth allowed to conduct a controlled comparison by measuringdeviations from it for each package. The ground truth here acts as a reference frame, to compare one software against another, and as such agreement or disagreement with its border definition is irrelevant.

### Parcels, metrics and statistical analysis

#### Package parcels

The cortical parcellation protocols, and in turn the derived parcels, differed across the 3 packages. We assessed parcels generated by FreeSurfer’s 2 latest and most suitable protocols for cortical analysis: the Desikan-Killiany (DK, [27]) and the Desikan-Killiany-Tourville (DKT, [19]) protocols. The DKT protocol was introduced in version 5.3 as an improvement on the DK protocol, offering better parcellation accuracy, clarity and consistency. BrainSuite parcellations are based on an adaptation of the LONI curve protocol [31], whereas the BrainGyrusMapping parcellations are done according to Neuromorphometrics’ brainCOLOR whole-brain protocol [28].

We focused our package analysis on 3 regions per subject hemisphere: the superior frontal gyrus (SFG) of the frontal lobe, the supramarginal gyrus (SMG) of the parietal lobe, and the cingulate gyrus (CG) of the cingulate cortex. These gyri were chosen on the basis that they are situated in different lobes, undergo structural changes with ageing [32] and dementia [33–37], and exhibit gender differences [32, 38, 39]. As the parcellation protocols differed, it was necessary at times to combine some parcels to produce comparable regions. Table 2 details the parcels we combined in each software package.

**Table 2:** A summary of the parcels we combined in each software package to yield comparable SFGs, SMGs and CGs

Software Package	SFG equivalent	SMG equivalent	CG equivalent
FreeSurfer-DK	SFG	SMG	rostral anterior cingulate + caudal anterior cingulate + posterior cingulate + isthmus cingulate
FreeSurfer-DKT	SFG	SMG	rostral anterior cingulate + caudal anterior cingulate + posterior cingulate + isthmus cingulate
BrainSuite	SFG	SMG	CG
BrainGyrusMapping	superior frontal gyrus medial segment (MSFG) + superior frontal gyrus	SMG	anterior (ACgG) + middle (MCgG) + posterior cingulate gyri (PCgG)

*FreeSurfer-DK* FreeSurfer parcellation according to the Desikan-Killiany protocol, *FreeSurfer-DKT* FreeSurfer parcellation according to the Desikan-Killiany-Tourville protocol

#### Reference parcels

The 10 subjects’ corresponding ground truth SFG, SMG and CG parcels which we compared to the package-derived parcels were manually segmented as described in [21]. This study’s source data and derivatives, including the left and right gyral parcels, are available in the Edinburgh DataShare repository [22].

#### Metrics and statistical analysis

Various metrics are automatically calculated by each of the tools. We chose the 3 most popular and relevant ones for our ageing population: grey matter thickness (GM_th_, e.g., [32–34, 40, 41]), grey matter volume (GM_vol_, e.g., [41, 42]), and white matter surface area (WM_sa_, e.g., [41, 42]). Both FreeSurfer and BrainSuite calculate these 3 metrics whilst BrainGyrusMapping provides GM_vol_ only. Several parcels were combined to form a region of interest depending on the region and package considered (Table 2). Metrics for such regions were derived by combining the original parcels’ metrics. For the case of GM_th_, this meant averaging individual parcel metrics, and for the case of GM_vol_ and WM_sa_, this meant adding individual metrics.

Statistical analyses consisted of (i) descriptive statistics (medians and 95% Bayesian highest density intervals (HDIs) for each metric, region of interest (ROI), and hemisphere and (ii) a percentile bootstrap between packages on relative median differences. Here the ground truth values are subtracted from each measure, and those measures are then compared across packages. This enables us to compare packages relative to a common reference. The percentile bootstrap was adjusted for multiple comparisons per metric (i.e. all measurements for each hemisphere/ROI included in a single procedure to maintain the type 1 error at 5% [43]). The raw data (tsv files) and the Matlab script we wrote to perform the data analysis are available in the Edinburgh DataShare repository [44].

## Results

Repeatability was observed for all packages, with identical results generated for each of the 3 runs (see tsv files of the Edinburgh DataShare repository [44]). Parcellation influences were also evident visually. We highlighted them using screenshots taken from various angles (see Additional file 1). We identified 6 double CG occurrences in this dataset: 4 in the left hemisphere (subjects 1, 5, 6 and 8) and 2 in the right hemisphere (subjects 6 and 10).

### Cortical volumes

**Fig. 1:**
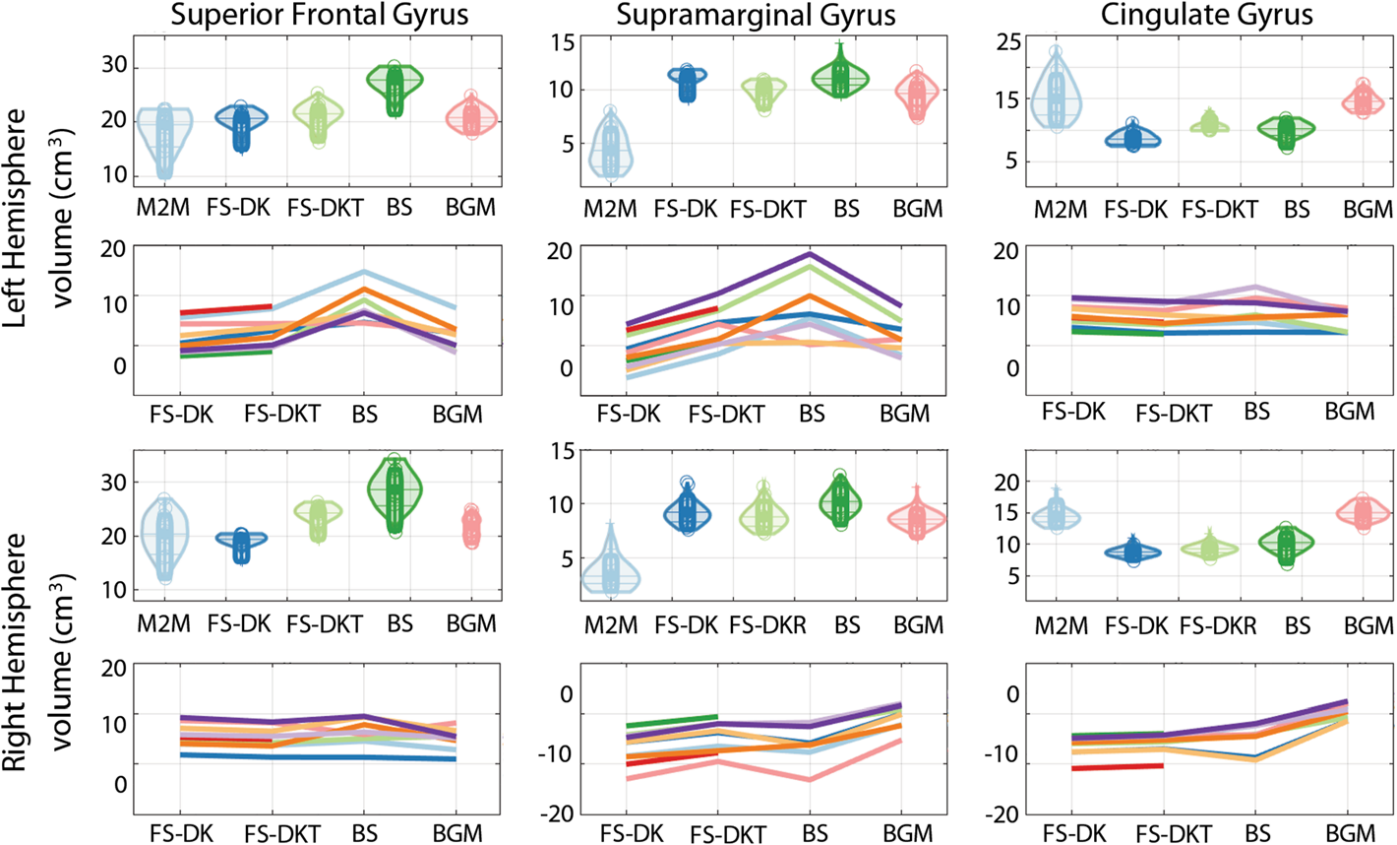
Violin plots show ROI cortical volume in cm^3^ computed by Masks2Metrics (M2M), FreeSurfer (FS-DK, FS-DKT), BrainSuite (BS), and BrainGyrusMapping (BGM) (the middle lines represent the medians, boxes the 95% Bayesian confidence intervals, and the density of the random average shifted histograms). Line plots show the relative difference from each package (FS, BS, BGM) to the ground truth estimates (M2M) for each subject (each line is a subject). Double CG occurrences were observed for subjects 1, 5, 6, and 8 in the left hemisphere, and subjects 6 and 10 in the right hemisphere. BrainSuite failed for subjects 4 and 6

Gray matter volumes automatically computed with the different packages were comparable, with overlapping confidence intervals (Fig. 1, Table 3) Compared to our ground truth, automated packages’ median volumes were all significantly higher for the SMG and all slightly larger for the SFG although not significantly different (overlap of confidence intervals). This difference in SFG is reflected by the smaller estimates seen for the neighbouring CG parcel (non-overlap of confidence intervals for FreeSurfer and BrainSuite, but not BGM).

**Table 3:** The median and HDIs (in mm^3^) for the cortical volume (GM_vol_) measurements

	Masks2Metrics Median [HDI]	FreeSurfer: DK atlas Median [HDI]	FreeSurfer: DKT atlas Median [HDI]	BrainSuite Median [HDI]	BrainGyrusMapping Median [HDI]
SFG					
left	19,503.53 [9913.7722069.11]	20,681.17 [14,686.9421537.14]	21,527.08 [16,623.7723099.58]	27,814.67 [21,414.3629212.03]	20,800.43 [17,852.8722681.01]
right	20,403.20 [12,194.7324057.71]	19,429.79 [15,404.2820261.04]	24,294.94 [19,114.7625609.17]	28,678.47 [20,745.3232308.96]	21,905.03 [18,689.6424212.91]
SMG					
left	4403.66 [2060.436335.99]	11,052.96 [9176.3911591.67]	10,019.87 [8209.6310722.12]	11,039.56 [9427.6712653.29]	9651.08 [7464.7110585.03]
right	3278.82 [1949.665159.66]	9220.52 [7603.2710626.95]	8800.85 [7220.0810266.21]	10,230.21 [8121.5411786.68]	8569.32 [6861.259386.06]
CG					
left	15,003.04 [10,855.2618826.12]	8553.84 [7541.809727.80]	10,552.95 [9925.1112077.79]	10,221.77 [7356.9711361.57]	14,587.58 [12,755.2016225.82]
right	14,447.00 [12,659.2516742.01]	8751.04 [7532.279607.27]	9275.60 [7665.5510144.70]	10,271.72 [6995.4111796.67]	15,036.93 [12,693.6516279.25]

*HDI* Highest density interval, *DK* Desikan-Killiany, *DKT* Desikan-Killiany-Tourville, *SFG* superior frontal gyrus, *SMG* supramarginal gyrus, *CG* cingulate gyrus

The comparison of relative median differences is shown in Table 4. Re-expressed in ground truth unit, most noticeable volume difference were observed for BrainSuite (which differed significantly from FreeSurfer for SFG volumes, and from BGM for the SFG and CG) and for BGM (which differed from all other packages for CG and from FreeSurfer for SFG). Looking at the subject’s plots (Fig. 1) reveals where differences are coming from. For the SMG volumes, larger differences were produced by BrainSuite. Its protocol vaguely defines the SMG, with only mention of it containing Brodmann area 40 and bordering the superior temporal gyrus [20, 31], hence the discrepancies within this package and across packages. For the CG volumes, when double gyri were present, they were not captured properly leading to underestimations, except for BGM especially in the right hemisphere. In addition, volume missing in the CG are sometimes misattributed to the SFG, in particular for BrainSuite. For instance, in subject 5, there is an omission of the upper CG fold caused by a double cingulate sulcus, making its SFG larger (see Additional file 1: Figure S1q-t). For subject 3 who has single CG occurrences, large relative SFG volumes are observed with BrainSuite because of differences in its medial, lateral and anterior borders compared to the remaining packages (indicated by arrows in (see Additional file 1: Figure S5 and S9)). Of interest, FreeSurfer DKT generates smaller relative volumes than DK for all CG scenarios (Fig. 1) because DKT accounts better than DK for double cingulate gyri, although imperfectly (Additional file 1: Figure S1, S2, S5, and S6). Furthermore, DKT’s relative SFG volumes are larger than DK’s for all subjects even when they are adjoining double CGs. Although the SFG in such cases loses its medial-most fold to the CG, with the DKT protocol the SFG is larger both anteriorly and posteriorly (i.e., lengthwise to include the majority of the frontal pole) as well as laterally, into the middle frontal gyrus, due to its revised border definitions [19]. This is evident pictorially in Additional file 1: Figure S1, S2, S5, S6, S9, S10, S11, and S12.

**Table 4:** Median GM_vol_ and confidence intervals (in mm^3^) differences between the packages relative to Masks2Metrics

	DK vs DKT	DK vs BS	DK vs BGM	DKT vs BS	DKT vs BGM	BS vs BGM
SFG_l						
Mdn	− 1255.57	− 7455.64	− 1038.89	− 6138.14	357.69	6497.02
CI	[− 1673.79–861.35]	[− 9813.19–4232.67]	[− 2185.45117.12]	[− 8657.26–2474.29]	[− 579.311108.25]	[3235.118427.86]
p	0.001*	0.001*	0.074	0.001*	0.438	0.001*
SFG_r						
Mdn	− 4863.43	− 9978.70	− 3356.71	− 5205.77	1739.75	6562.27
CI	[− 5423.49–4214.39]	[− 13,053.65–5992.21]	[− 3976.01–2306.43]	[− 8251.40–743.62]	[763.892702.32]	[2187.239684.41]
p	0.001*	0.001*	0.001*	0.018*	0.001*	0.001*
SMG_l						
Mdn	834.74	−5.13	1332.18	− 952.46	374.88	1544.30
CI	[698.641110.56]	[− 1528.761019.82]	[273.012264.50]	[− 2288.88 45.24]	[− 704.361532.69]	[− 135.153192.02]
p	0.001*	0.948	0.016*	0.076	0.546	0.072
SMG_r						
Mdn	419.04	− 504.81	602.52	− 957.20	171.20	1476.29
CI	[345.40520.80]	[− 1788.78641.65]	[−77.551141.13]	[− 2317.49135.09]	[− 525.82728.70]	[−223.252901.30]
p	0.001*	0.222	0.08	0.064	0.546	0.084
CG_l						
Mdn	− 2200.11	− 1257.29	− 6072.94	1002.98	− 3785.19	− 4852.73
CI	[− 2550.00–1915.54]	[− 2281.19128.11]	[− 6423.74–5459.66]	[−113.802519.34]	[− 4245.08–3252.80]	[− 6245.32–3756.85]
p	0.001*	0.084	0.001*	0.084	0.001*	0.001*
CG_r						
Mdn	− 506.70	− 1529.36	− 6166.02	− 1063.50	− 5657.51	−4852.15
CI	[− 578.10–415.37]	[− 2542.65430.84]	[− 6644.40–5882.54]	[− 2051.44992.76]	[− 6162.33–5373.53]	[− 6851.68–3668.69]
p	0.001*	0.126	0.001*	0.254	0.001*	0.001*

*DK* Desikan-Killiany, *DKT* Desikan-Killiany-Tourville, *BS* BrainSuite, *BGM* BrainGyrusMapping, *SFG_l/SFG_r* left/right superior frontal gyrus, *SMG_l/SMG_r* left/right supramarginal gyrus, *CG_l/CG_r* left/right cingulate gyrus, *Mdn* median, *CI* confidence interval, *: significant difference

### Cortical thickness

**Fig. 2:**
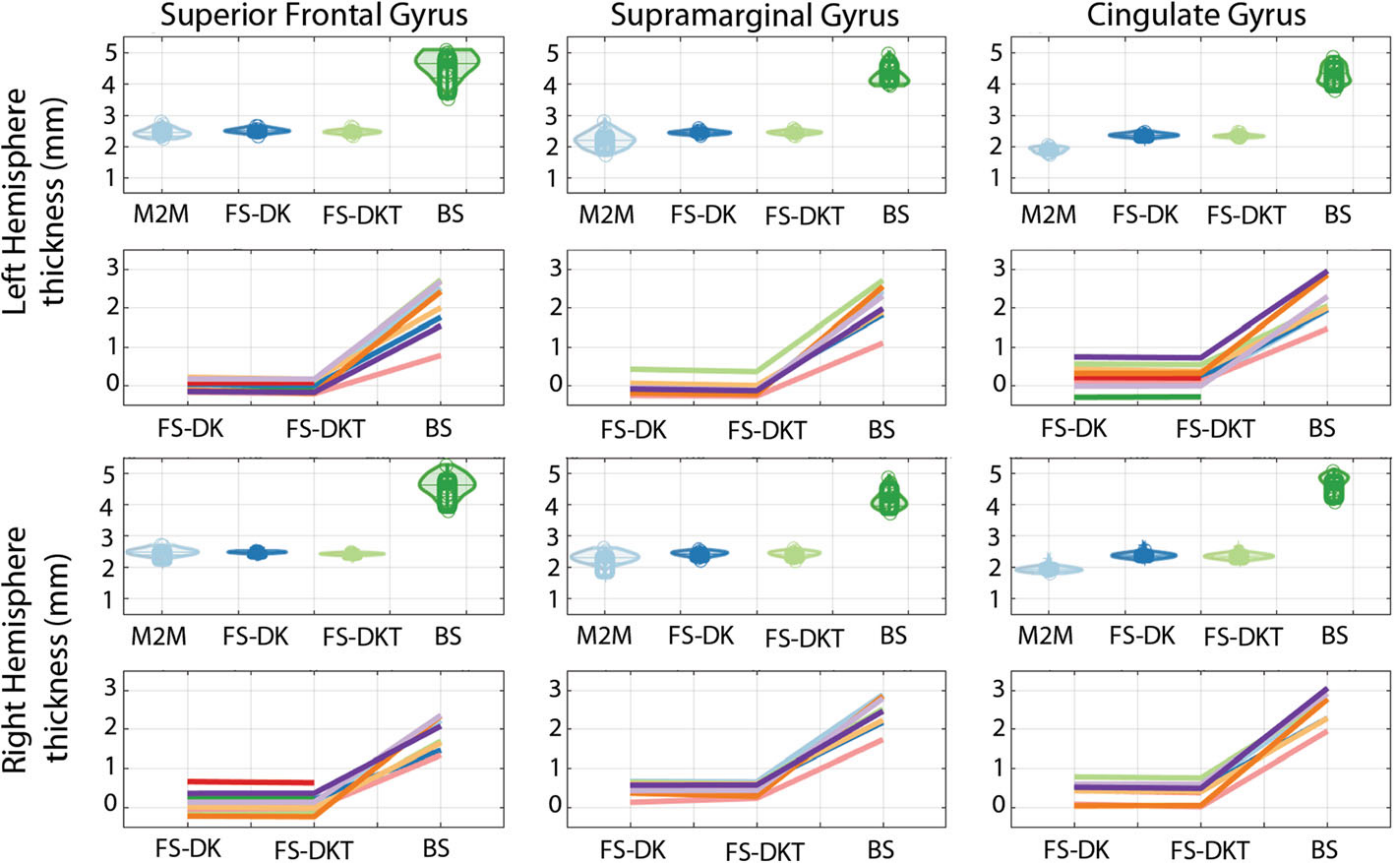
Violin plots show ROI cortical thickness in mm computed by Masks2Metrics (M2M), FreeSurfer (FS-DK, FS-DKT), and BrainSuite (BS) (the middle lines represent the medians, boxes the 95% Bayesian confidence intervals, and the density of the random average shifted histograms). Line plots show the relative difference from each package (FS, BS) to the ground truth estimates (M2M) for each subject (each line is a subject). Double CG occurrences were observed for subjects 1, 5, 6, and 8 in the left hemisphere, and subjects 6 and 10 in the right hemisphere. BrainSuite failed for subjects 4 and 6

Cortical thickness measurements computed following FreeSurfer’s two parcellation routes were very similar to the ground truth (overlap of 95% HDI) while BrainSuite show significantly higher estimate than all other packages (just under double those of the other methods) along with higher dispersion (Fig. 2, Table 5). All packages were, however, still in agreement with the reported post-mortem values taken at the lateral (3.5 mm), medial (2.7 mm) and overall (2.5 mm) cortical surfaces [45].

**Table 5:** Median and HDIs (in mm) for cortical thickness measurements

ROI	Masks2Metrics Median [HDI]	FreeSurfer: DK atlas Median [HDI]	FreeSurfer: DKT atlas Median [HDI]	BrainSuite Median [HDI]
SFG				
left	2.46 [2.26 2.63]	2.51 [2.34 2.60]	2.48 [2.34 2.56]	4.65 [3.51 4.99]
right	2.50 [2.12 2.63]	2.49 [2.36 2.52]	2.43 [2.30 2.46]	4.64 [3.80 4.94]
SMG				
left	2.20 [1.75 2.45]	2.45 [2.35 2.52]	2.45 [2.34 2.51]	4.30 [3.95 4.72]
right	2.32 [1.72 2.48]	2.44 [2.24 2.50]	2.43 [2.23 2.51]	4.23 [3.73 4.69]
CG				
left	1.89 [1.74 1.98]	2.37 [2.18 2.45]	2.35 [2.29 2.42]	4.33 [3.79 4.70]
right	1.95 [1.82 2.09]	2.40 [2.23 2.52]	2.37 [2.15 2.52]	4.62 [4.09 4.94]

*HDI* Highest density interval, *DK* Desikan-Killiany, *DKT* Desikan-Killiany-Tourville, *ROI* region of interest, *SFG* superior frontal gyrus, *SMG* supramarginal gyrus, *CG* cingulate gyrus

Relative to the ground truth, BrainSuite showed a significant difference to both FreeSurfer outputs (DK and DKT) for all ROIs (Table 6). Examination of differences per subject (Fig. 2) revealed little difference between DK and DKT, yet large differences between them and BrainSuite, as well as across subjects within BrainSuite. This is explained (i) by the fact that thickness is not expected to change at the borders of parcels, and therefore differences in volume between DK and DKT do not translate into differences in thickness and (ii) BrainSuite combines grey and white matter thicknesses rather than just grey matter (see Discussion).

**Table 6:** Median GM_th_ and confidence intervals (in mm) differences between the packages relative to Masks2Metrics

	Left hemisphere			Right hemisphere		
	DK vs DKT	DK vs BS	DKT vs BS	DK vs DKT	DK vs BS	DKT vs BS
SFG						
Mdn [CI]	0.04[0.01 0.06]	−2.09[−2.50–1.63]	−2.13[−2.54–1.66]	0.05[0.04 0.06]	− 2.15[− 2.44–1.80]	−2.20[− 2.49–1.85]
p	0.001*	0.001*	0.001*	0.001*	0.001*	0.001*
SMG						
Mdn [CI]	0.00[−0.01 0.02]	−1.86[−2.25–1.56]	−1.86[− 2.24–1.57]	0.01[0.00 0.02]	−1.80[− 2.17–1.49]	−1.81[− 2.18–1.48]
p	0.76	0.001*	0.001*	0.18	0.001*	0.001*
CG						
Mdn [CI]	0.01[−0.01 0.04]	−1.91[− 2.27–1.65]	−1.93[− 2.28–1.65]	0.03[0.01 0.03]	−2.12[− 2.44–1.88]	−2.15[− 2.45–1.93]
p	0.18	0.001*	0.001*	0.001*	0.001*	0.001*

*SFG* superior frontal gyrus, *SMG* supramarginal gyrus, *CG* cingulate gyrus, *Mdn* median, *CI* confidence interval, *: significant difference

### Surface area

**Fig. 3:**
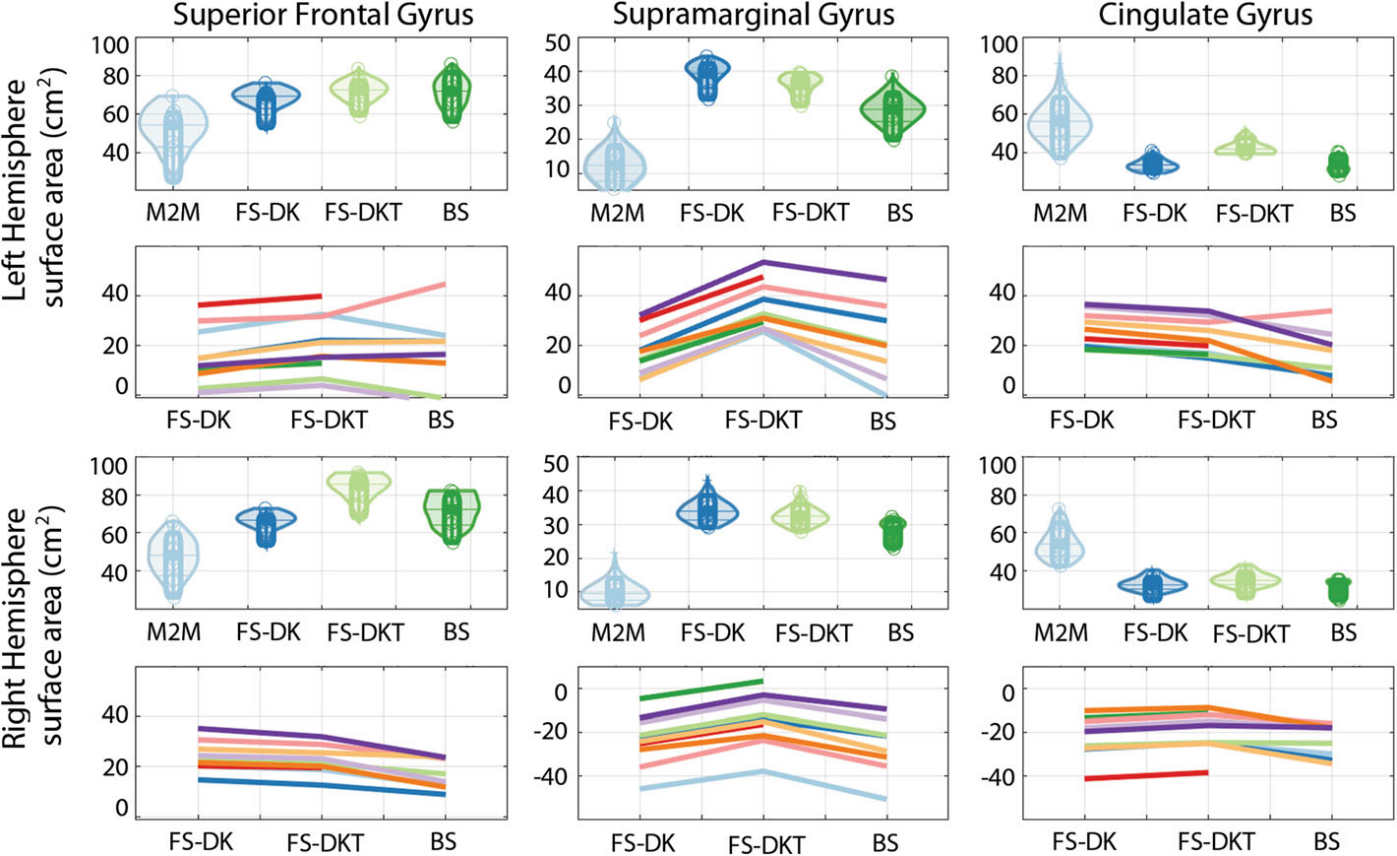
Violin plots show ROI cortical surface area in mm^2^ computed by Masks2Metrics (M2M), FreeSurfer (FS-DK, FS-DKT), and BrainSuite (BS) (the middle lines represent the medians, boxes the 95% Bayesian confidence intervals, and the density of the random average shifted histograms). Line plots show the relative difference from each package (FS, BS) to the ground truth estimates (M2M) for each subject (each line is a subject). Double CG occurrences were observed for subjects 1, 5, 6, and 8 in the left hemisphere, and subjects 6 and 10 in the right hemisphere. BrainSuite failed for subjects 4 and 6

The packages’ SFG and SMG surface area metrics were generally larger than the ground truth, whereas their CG metrics were generally smaller (Fig. 3, Table 7).

**Table 7:** Median and HDIs (in mm^2^) for the surface area (WM_sa_) measurements

	Masks2Metrics Median [HDI]	FreeSurfer: DK atlas Median [HDI]	FreeSurfer: DKT atlas Median [HDI]	BrainSuite Median [HDI]
SFG				
left	5418.63[2524.486077.87]	6932.32[5241.287162.24]	7251.39[5914.927728.96]	7184.29[5600.438178.45]
right	4821.43[2678.175897.71]	6666.56[5371.966969.92]	8553.63[6779.629010.75]	7234.76[5492.588031.08]
SMG				
left	1238.45[472.231741.25]	3945.28[3211.174204.30]	3610.66[2979.003898.30]	2884.20[1980.263320.66]
right	951.54[606.111403.36]	3390.12[2914.763794.86]	3244.95[2788.073609.39]	2806.16[2283.533103.18]
*CG*				
left	5593.20[3681.956780.48]	3342.94[2928.283721.53]	4169.58[3917.894699.32]	3354.73[2778.523907.92]
right	5411.63[4339.366527.50]	3261.92[2445.713667.55]	3499.70[2602.553924.99]	3156.88[2448.173521.72]

*HDI* highest density interval, *DK* Desikan-Killiany, *DKT* Desikan-Killiany-Tourville, *SFG* superior frontal gyrus, *SMG* supramarginal gyrus, *CG* cingulate gyrus

Relative to the ground truth, all SMG measurements were significantly different to one another in both hemispheres (Table 8). Significant differences existed between DKT and the remaining methods for all ROIs except for the left SFG when compared to BrainSuite). As with the relative cortical volumes, the largest relative surface areas were generally in the subjects with the double CG occurrence at both the CG and the affected SFG because larger gyral volumes are expected to have larger surface areas. Once again, DKT generated smaller relative volumes than DK for all CG scenarios as it accounted better than DK of both single and double gyri (see Additional file 1: Figure S1, S2, S5, and S6). Unlike other subjects, subject 5’s left SMG surface area with BrainSuite is relatively larger than its equivalent in the remaining protocols. This is also evident pictorially (see Additional file 1: Figure S3q-t) which demonstrates a wider BrainSuite SMG, terminating caudally, like DK, at the second segment of the caudal superior temporal sulcus rather than at the first segment as with DKT and BrainGyrusMapping.

**Table 8:** Median WM_sa_ differences (in mm^2^) between packages relative to the ground truth

	Left hemisphere			Right hemisphere		
	DK vs DKT	DK vs BS	DKT vs BS	DK vs DKT	DK vs BS	DKT vs BS
SFG						
Mdn[CI]	− 435.36 [−667.46–293.24]	−352.74[− 789.72219.10]	235.96[− 304.49679.00]	− 1878.16[− 2023.98–1702.11]	−665.24[− 116.37 24.94]	1172.29[848.961796.02]
p	0.001*	0.236	0.298	0.001*	0.06	0.001*
SMG						
Mdn[CI]	300.02[253.18383.49]	1164.67[649.751554.81]	810.26[381.131236.37]	146.45[125.05193.34]	781.26[555.911007.28]	612.97[401.72817.49]
p	0.001*	0.001*	0.004*	0.001*	0.001*	0.001*
CG						
Mdn[CI]	− 899.53[− 1009.71–819.93]	45.99[− 165.47365.27]	990.09[796.881223.38]	−247.98[− 285.87–192.15]	230.25[−48.94616.47]	490.93[183.26830.57]
p	0.001*	0.84	0.001*	0.001*	0.116	0.001*

*DK* Desikan-Killiany, *DKT* Desikan-Killiany-Tourville, *BS* BrainSuite, *SFG* superior frontal gyrus, *SMG* supramarginal gyrus, *CG* cingulate gyrus, *Mdn* median, *CI* confidence interval, *: significant difference

## Discussion

The parcellation protocol we followed while segmenting the ground truth parcels enabled us to consistently identify and address any visible anatomical variability (see Additional file 1, [21]). Because of this, the parcels’ shapes varied greatly across the cohort, creating large dispersions in the ground truth volumes (Fig. 1) and surface areas (Fig. 3). Using this as a reference frame to compare packages allowed thus to highlight how each package deals with these natural variations. The main contributor to variability in the CG and SFG is the cingulate sulcus [46] which can have a single or double occurrence (and therefore a double CG occurrence), branches, as well as discontinuities, all of which are interpreted differently by each package. Given that it defines the dividing landmark between the CG and SFG, both gyri are highly variable, as are their volumes and surface areas. The SMG is also highly variable across the cohort, mainly due to its posterior border, as is its segmentation across the packages.

**Fig. 4:**
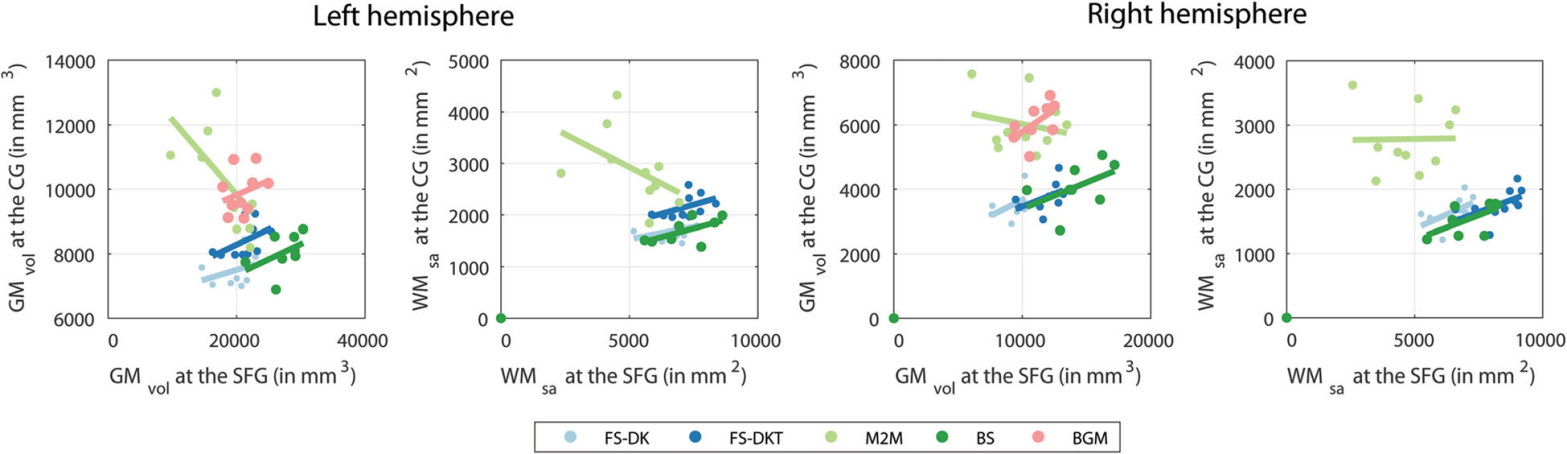
Correlations between SFG GMvol and WMsa with CG GMvol and WMsa for the ground truth (M2M) and parcels obtained automatically

The size of our dataset and the use of 1.5 T MRI images are of course a limitation. There are variations which depends on age (in adults) that would be better captured with a larger sample capturing a wider range of age and higher resolution images. This is particularly true for gyrification (the process and the extent of folding) which varies with age [5] and can thus impact on the identification of anatomical branches and borders. The current dataset was nevertheless variable enough to highlight issues in automated packages. For what is reported here, i.e. that the differences observed mainly stem from how anatomical variability in additional gyri and branching is handled, aging or higher resolution imaging has no impact. For instance, the presence/absence of double gyri is observed once the brain is fully formed and does not change across adulthood and is observed even with coarse image resolution.

With volume being (in theory) a product of thickness and surface area, and the thicknesses being generally stable for each package, larger surface areas are expected to accompany larger volumes, and vice versa and this is what we saw. We also observed that the inability to fully capture anatomical variability has knock-on effects on neighbouring regions, as was the case in FreeSurfer, BrainSuite, and BrainGyrusMapping where SFG GM_vol_ and WM_sa_ are proportional to the CG GM_vol_ and WM_sa_, whilst no or the reverse effect were observed when segmenting regions manually (Fig. 4).

Although our work highlights differences between parcellation protocols, it is most likely that the corresponding outputs of image analysis tools in fact vary due to a combination of factors, and not just the parcellation phase. One step prior to parcellation in automated and semi-automated tools is the pre-processing phase. In FreeSurfer, for example, amongst other things, that phase is used to derive white and grey matter masks [1]. These are consequently split in the processing stage, as per a parcellation protocol, to form parcels. Such mask effects were not investigated in this manuscript although it could be contributing to differences, especially for thickness. Package inconsistency across sites (e.g., [47]) and operating systems (e.g., [16]) is another aspect to consider, although was not a contributing factor to our study as each package was run on only one computer and one operating system. Finally, and most relevant here, differences in algorithms can also account for observed differences. Volume is simply derived by counting the number of voxels in each parcel and thus directly reflects differences in parcellation protocols. Cortical thickness however is specific to grey matter in FreeSurfer, while in BrainSuite it refers to that of the gyrus, all the way down to the fundus, therefore capturing the combined grey and white matter thicknesses [31]. The combination of parcel definition and using the sulcal fundus to mark the border of a gyrus also explains inconsistencies in surface area measurements.

## Conclusions

We previously investigated package differences in terms of their parcellation protocol definitions, raising awareness of the associated uncertainties stemming from the well-reported anatomical variability that they are likely to encounter [20]. In our present work, we quantify the effects of these uncertainties through a healthy middle-aged dataset and manually-derived ground truth data with associated morphometrics. We show that multi-atlas parcellation (BGM) is the most accurate method and therefore encourage more research and usage of such tools. Explicit definition of the method used to compute thickness and surface area is another major factor, and since multi-atlas methods are currently limited to volume, we recommend using FeeeSurfer’s DKT approach with manual editing to derive grey matter thickness and white matter surface area.

## Additional file

Additional file 1: Package screenshots. Screenshots from FreeSurfer, BrainSuite, and BrainGyrusMapping parcellation for each of the 10 subjects. The screenshots are occasionally overlaid by their equivalent ground truth parcellations. (DOCX 28298 kb)

## Abbreviations

ACgG: Anterior cingulate gyrus
BGM: BrainGyrusMapping
BGM: BrainGyrusMapping
BIDS: Brain Imaging Data Structure
BS: BrainSuite
CG: Cingulate gyrus
CG_l/CG_r: Left/right cingulate gyrus
CI: Confidence interval
DK: Desikan-Killiany
DKT: Desikan-Killiany-Tourville
FreeSurfer-DK or FS-DK: FreeSurfer parcellation according to the Desikan-Killiany protocol
FreeSurfer-DKT or FS-DKT: FreeSurfer parcellation according to the Desikan-Killiany-Tourville
GM_th_: Grey matter thickness
GM_vol_: Grey matter volume
HDI: Highest density interval
MCgG: Middle cingulate gyrus
Mdn: Median
MICCAI: Medical Image Computing and Computer Assisted Intervention
MRI: Magnetic resonance imaging
MSFG: Superior frontal gyrus medial segment
PCgG: Posterior cingulate gyrus
ROI: Region of interest
SFG: Superior frontal gyrus
SFG: Superior frontal gyrus
SFG_l /SFG_r: Left/right superior frontal gyrus
SMG: Supramarginal gyrus
SMG_l/SMG_r: Left/right supramarginal gyrus
T1w: T1-weighted
T2w: T2-weighted
WM_sa_: White matter surface area

## Footnotes

1 Formerly Toshiba Medical Visualization Systems Europe.
