# Supplementary material for: A controlled comparison of thickness, volume and surface areas from multiple cortical parcellation packages
Source: BMC Bioinformatics. 2019 Jan 28;20:55. doi: 10.1186/s12859-019-2609-8 (PMC6348615; doi:10.1186/s12859-019-2609-8)
Supplement: Supplementary file 1 — Package screenshots. Screenshots from FreeSurfer, BrainSuite, and BrainGyrusMapping parcellation for each of the 10 subjects. The screenshots are occasionally overlaid by their equivalent ground truth parcellations. (DOCX 28298 kb) [file 12859_2019_2609_MOESM1_ESM.docx]

Additional file 1

***
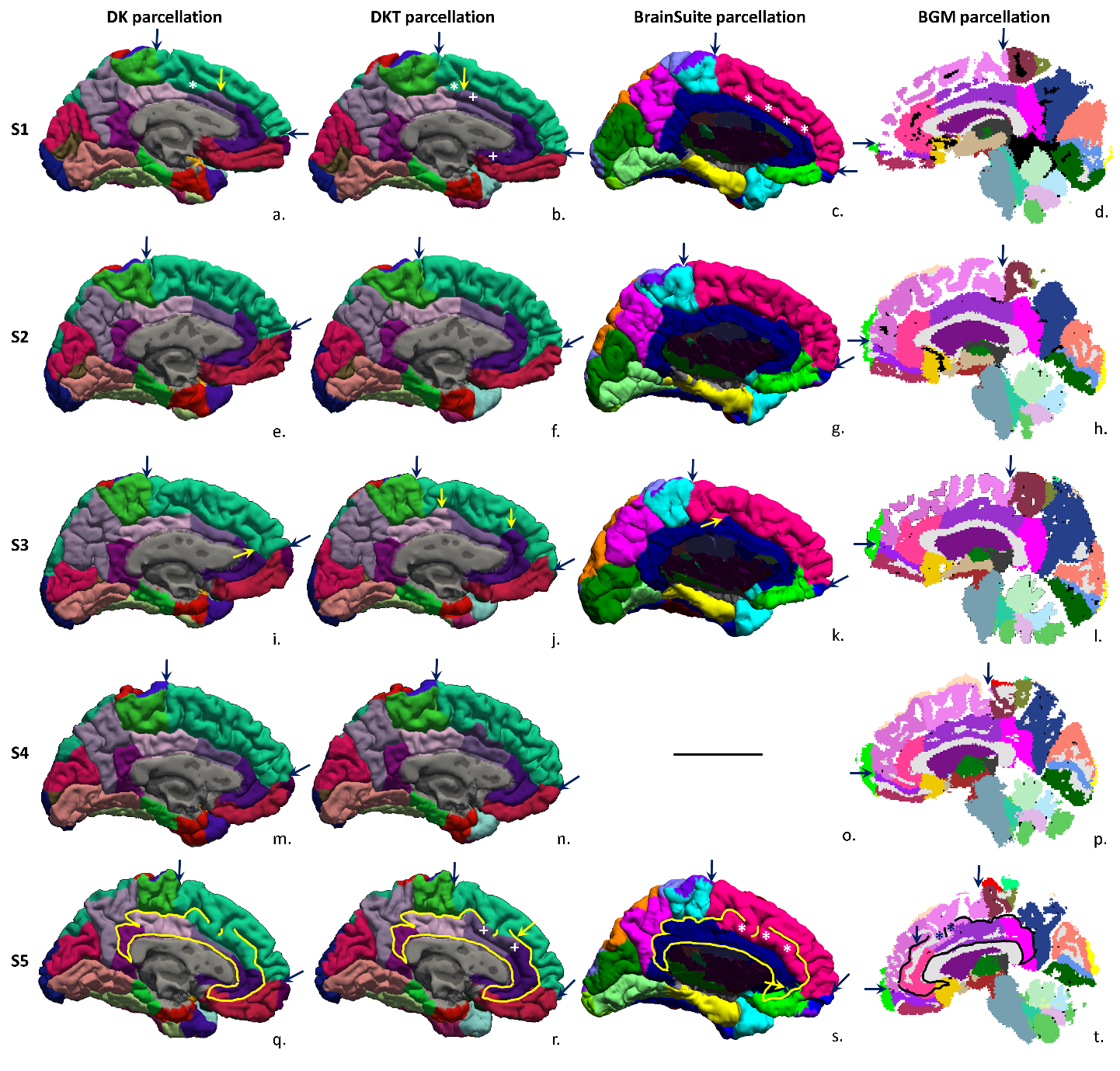
Figure S1.*** *Medial view of cortical parcellation for subjects 1-5’s left hemisphere. Parcellation was done according to FreeSurfer (DK and DKT protocols), BrainSuite, and BrainGyrusMapping. The variable anterior, posterior and medial SFG borders of each package are indicated by arrows. The ground truth CG is outlined on top of subject 5’s parcellations (q-t). DK: Desikan-Killiany; DKT: Desikan-Killiany-Tourville; BGM: BrainGyrusMapping; ‘+’: additional CG regions in DKT compared to DK; ‘*’: regions missing from the CG according to ground truth parcellation;.*

***
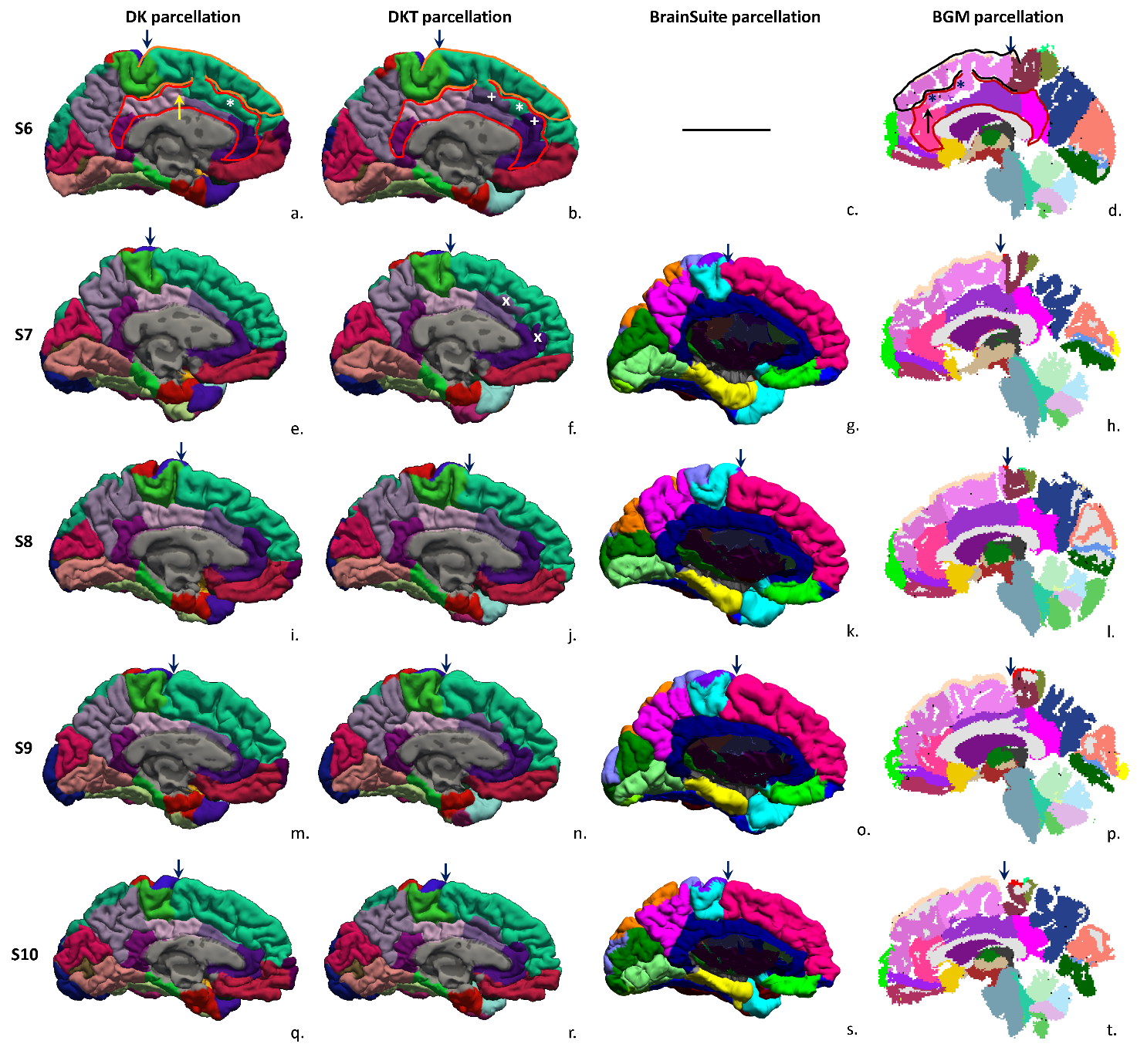
Figure S2.*** *Medial view of cortical parcellation for subjects 6-10’s left hemisphere. Parcellation was done according to FreeSurfer (DK and DKT protocols), BrainSuite, and BrainGyrusMapping. The variable posterior SFG borders of each package are indicated by arrows. The ground truth SFG and CG are outlined on top of subject 6’s parcellations (a-d). DK: Desikan-Killiany; DKT: Desikan-Killiany-Tourville; BGM: BrainGyrusMapping; ‘+’: additional CG regions in DKT compared to DK; ‘*’: regions missing from the CG according to ground truth parcellation; ‘x’: regions wrongly attributed to the CG, according to ground truth parcellation.*

***
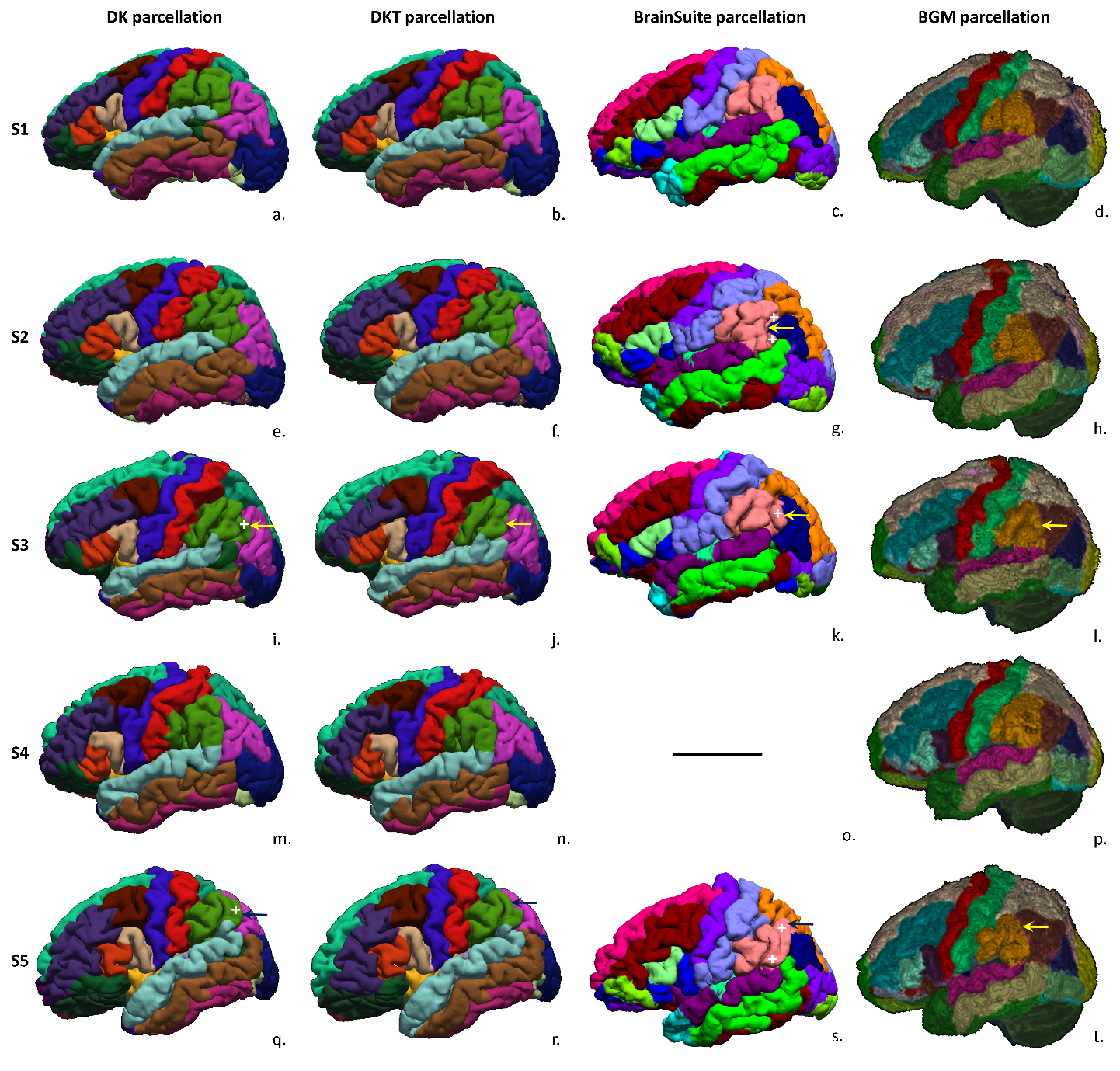
Figure S3.*** *Lateral view of cortical parcellation for subjects 1-5’s left hemisphere. Parcellation was done according to FreeSurfer (DK and DKT protocols), BrainSuite, and BrainGyrusMapping. The highly variable posterior SMG borders are indicated by arrows, with a ‘+’ representing the additional SMG folds of BrainSuite (g, k, s). DK: Desikan-Killiany; DKT: Desikan-Killiany-Tourville; BGM: BrainGyrusMapping.*

***
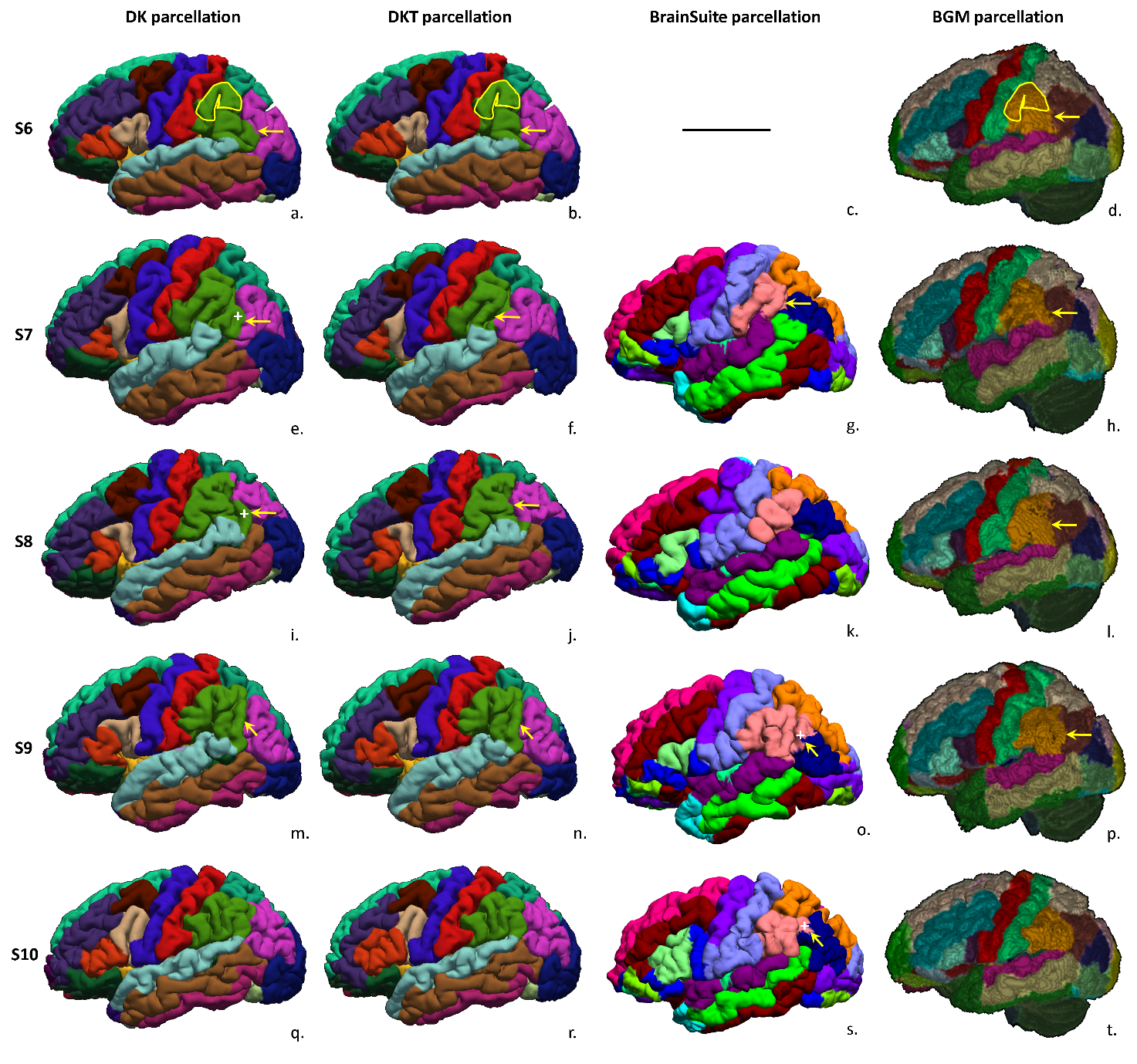
Figure S4.*** *Lateral view of cortical parcellation for subjects 6-10’s left hemisphere. Parcellation was done according to FreeSurfer (DK and DKT protocols), BrainSuite, and BrainGyrusMapping. The highly variable posterior SMG borders of each package are indicated by arrows. The ground truth SMG is outlined on top of subject 6’s parcellations (a-d). DK: Desikan-Killiany; DKT: Desikan-Killiany-Tourville; BGM: BrainGyrusMapping; ‘+’: additional SMG folds in the packages, compared to DKT.*

***
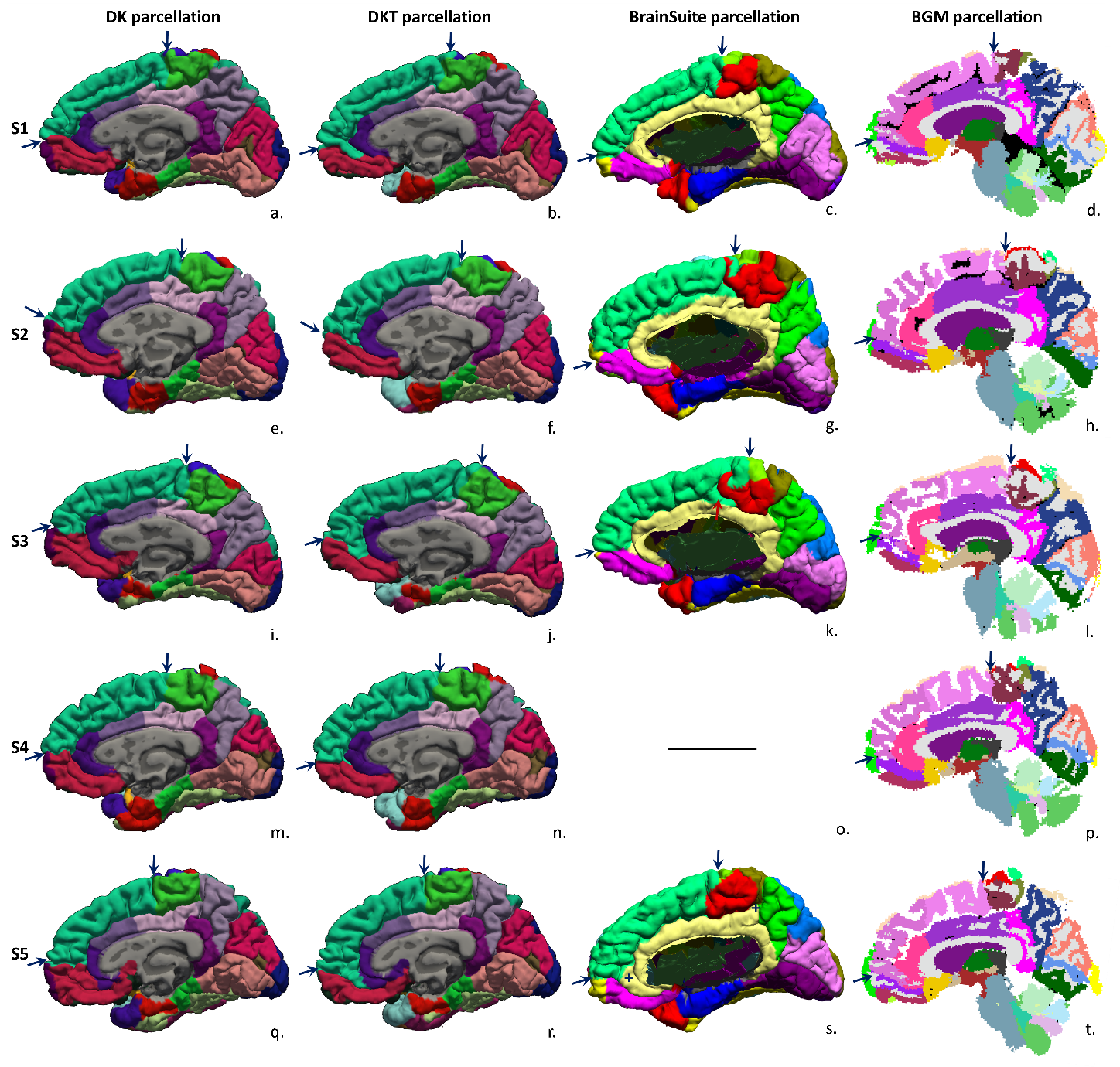
Figure S5.*** *Medial view of cortical parcellation for subjects 1-5’s right hemisphere. Parcellation was done according to FreeSurfer (DK and DKT protocols), BrainSuite, and BrainGyrusMapping. SFG borders for each of the packages are indicated by arrows. DK: Desikan-Killiany; DKT: Desikan-Killiany-Tourville; BGM: BrainGyrusMapping.*

*
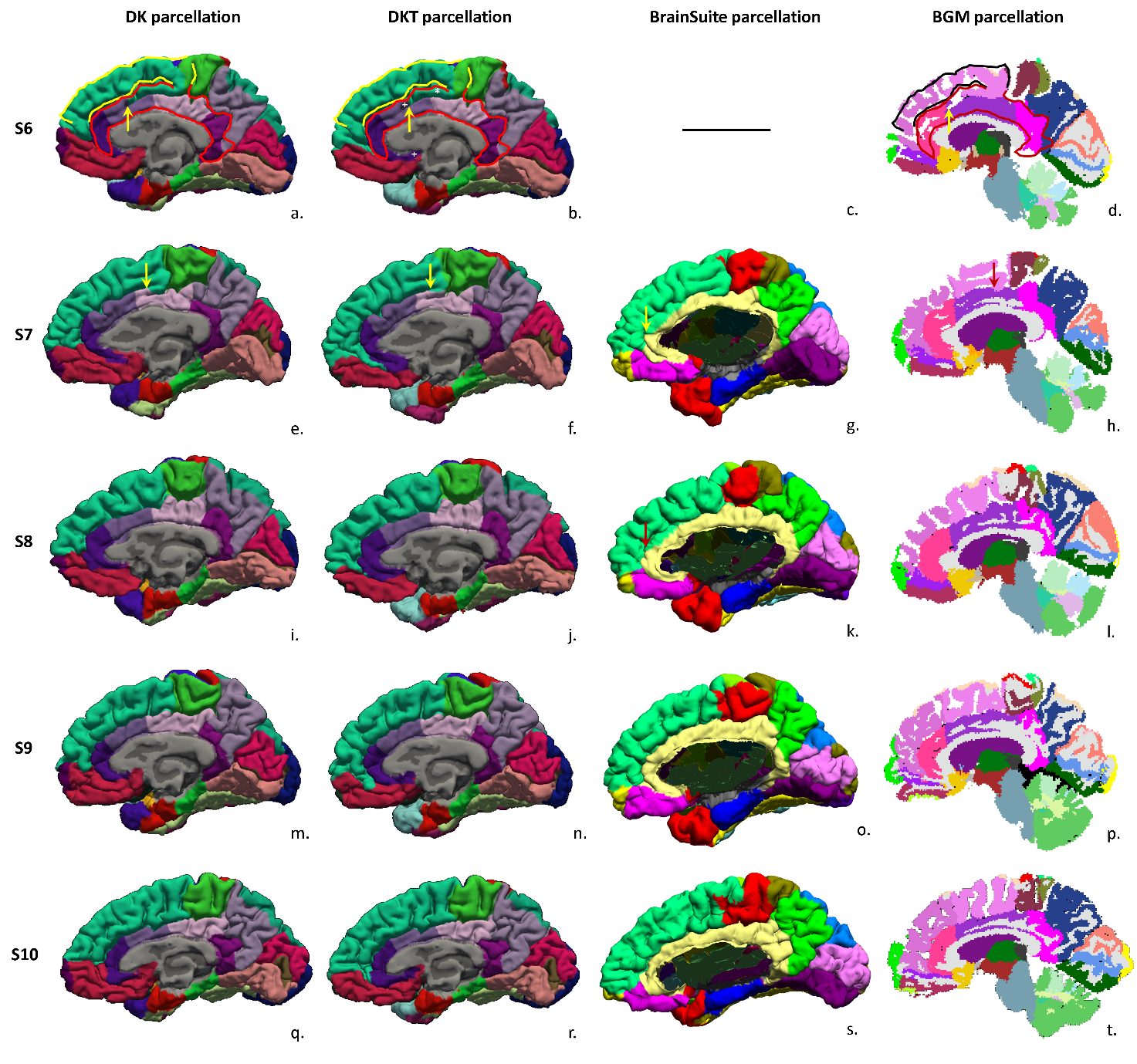
****Figure S6.*** *Medial view of cortical parcellation for subjects 6-10’s right hemisphere. Parcellation was done according to FreeSurfer (DK and DKT protocols), BrainSuite, and BrainGyrusMapping. Package discrepancies at the SFG-CG border are indicated by arrows. The ground truth SFG and double CG are outlined on top of subject 6’s parcellations (a-d). DK: Desikan-Killiany; DKT: Desikan-Killiany-Tourville; BGM: BrainGyrusMapping.*

***
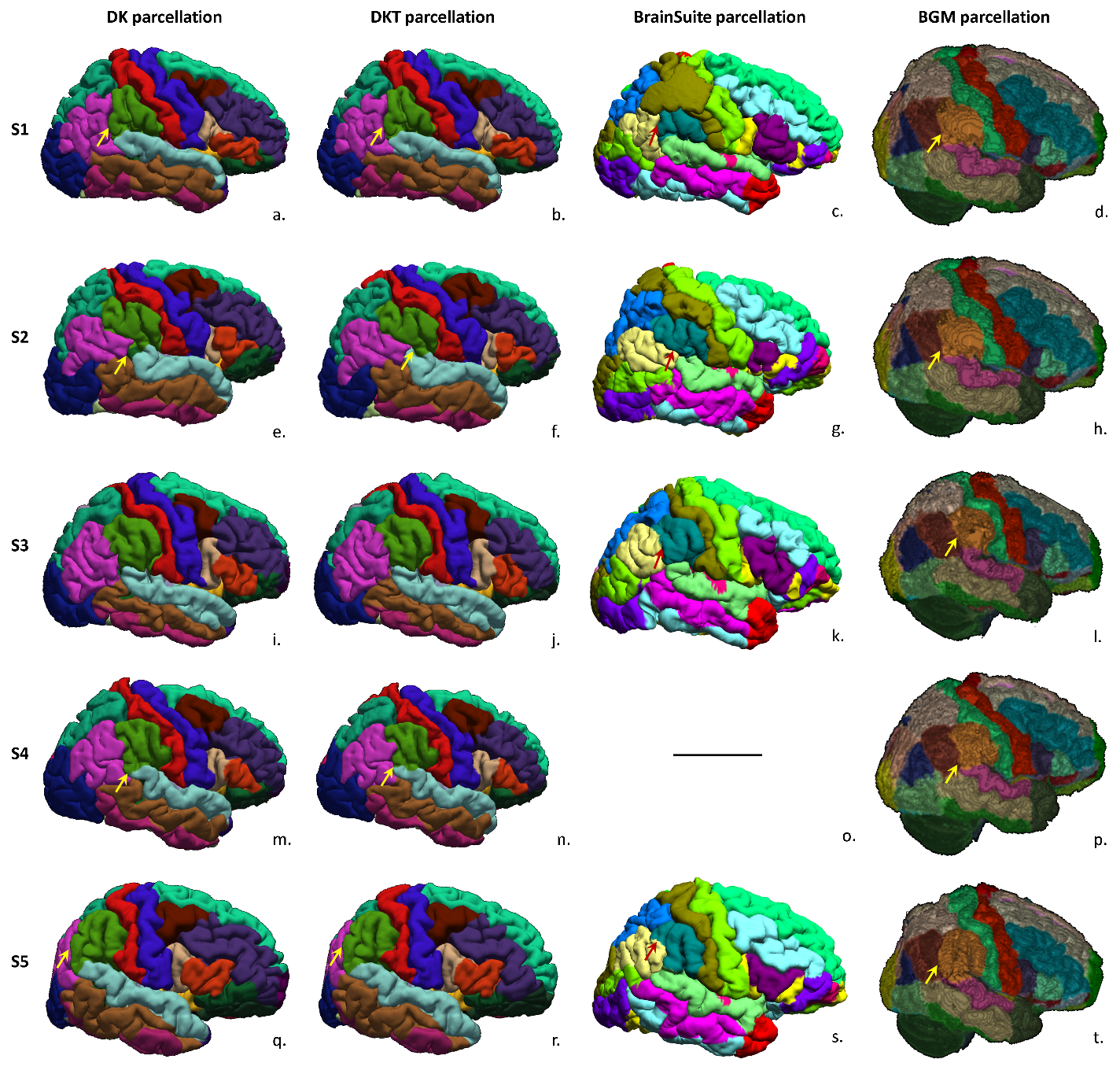
Figure S7.*** *Lateral view of cortical parcellation for subjects 1-5’s right hemisphere. Parcellation was done according to FreeSurfer (DK and DKT protocols), BrainSuite, and BrainGyrusMapping. Posterior SMG borders are indicated by arrows. DK: Desikan-Killiany; DKT: Desikan-Killiany-Tourville; BGM: BrainGyrusMapping.*

***
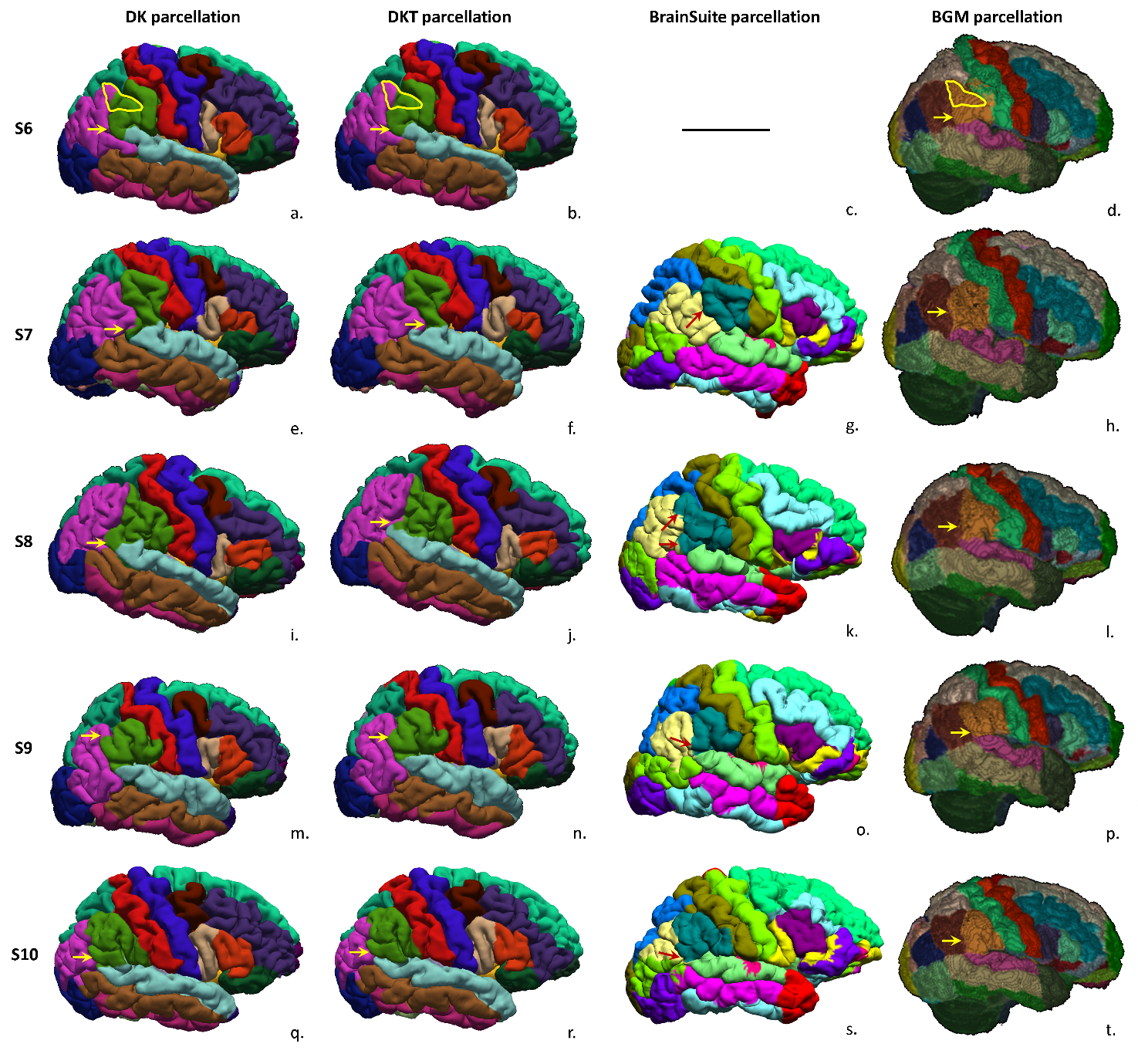
Figure S8.*** *Lateral view of cortical parcellation for subjects 6-10’s right hemisphere. Parcellation was done according to FreeSurfer (DK and DKT protocols), BrainSuite, and BrainGyrusMapping. Posterior SMG borders are indicated by arrows. The ground truth SMG is outlined on top of subject 6’s parcellations (a-d). DK: Desikan-Killiany; DKT: Desikan-Killiany-Tourville; BGM: BrainGyrusMapping.*

***
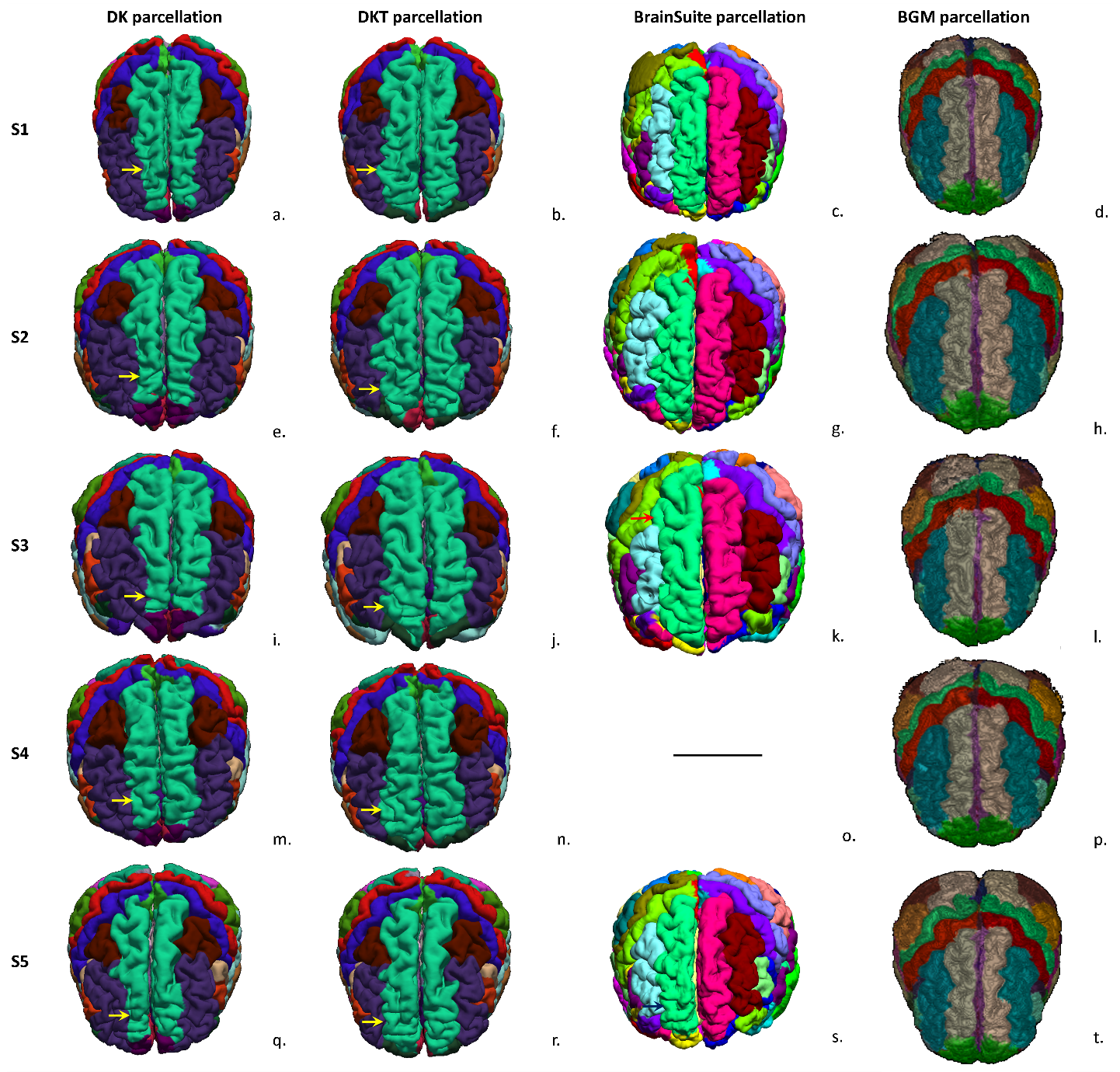
Figure S9.*** *Superior view of cortical parcellation for subjects 1-5. Parcellation was done according to FreeSurfer (DK and DKT protocols), BrainSuite, and BrainGyrusMapping. Differences between DK and DKT protocols at the antero-lateral end of the SFG are indicated by arrows. DK: Desikan-Killiany; DKT: Desikan-Killiany-Tourville; BGM: BrainGyrusMapping.*

***
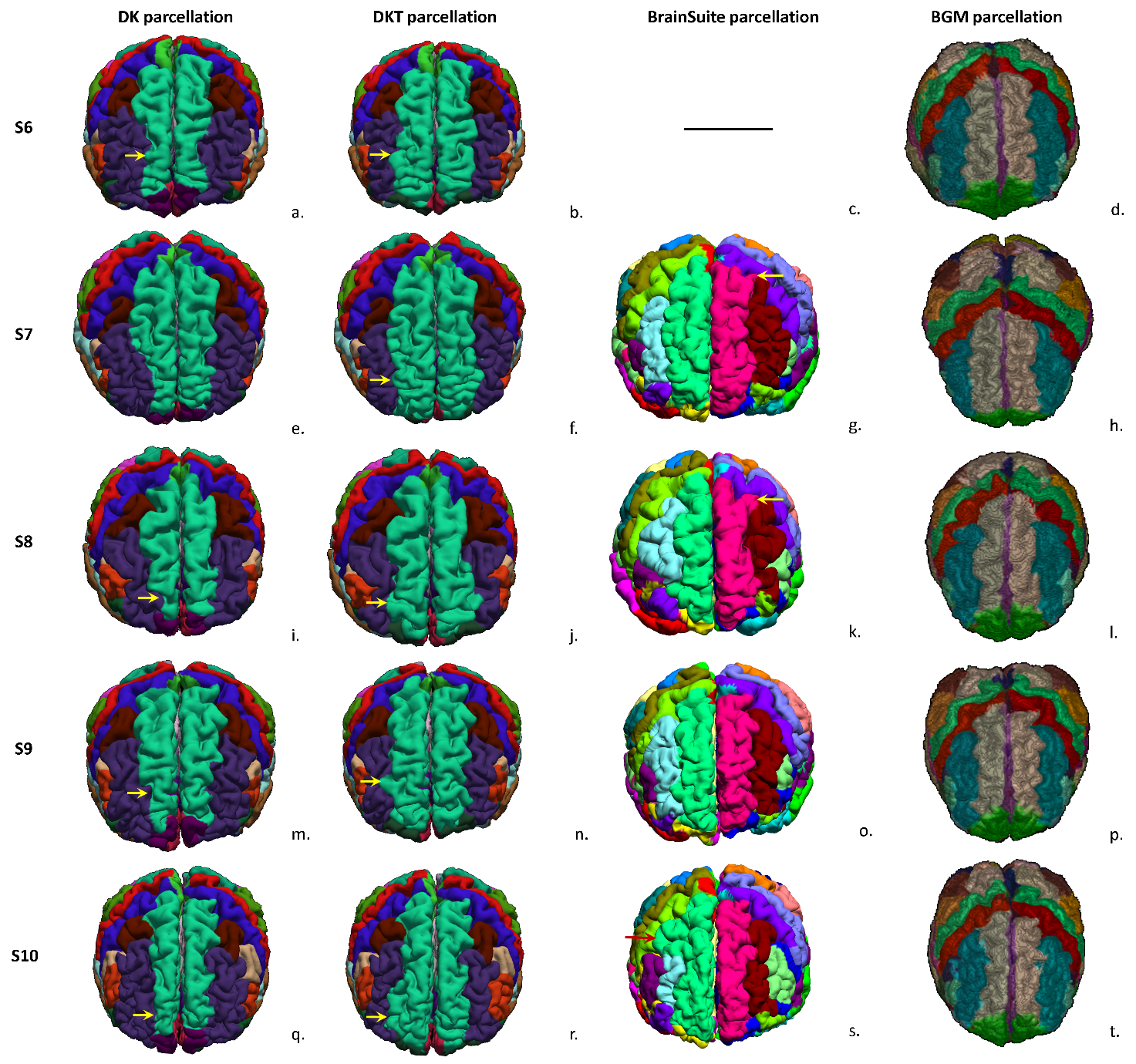
Figure S10.*** *Superior view of cortical parcellation for subjects 6-10. Parcellation was done according to FreeSurfer (DK and DKT protocols), BrainSuite, and BrainGyrusMapping. Protocol differences at the SFG are indicated by arrows. DK: Desikan-Killiany; DKT: Desikan-Killiany-Tourville; BGM: BrainGyrusMapping.*

***
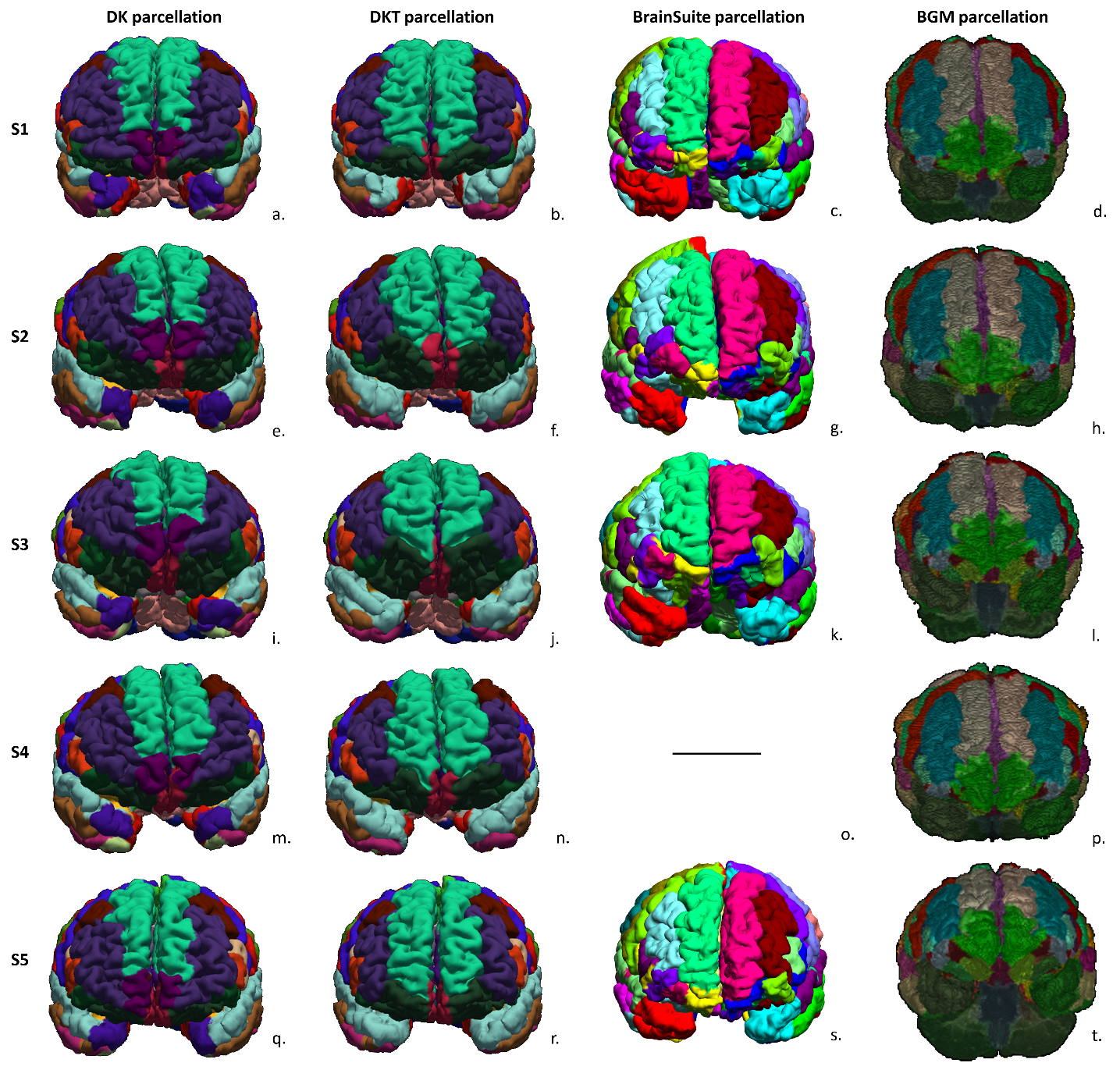
Figure S11.*** *Antero-superior view of cortical parcellation for subjects 1-5. Parcellation was done according to FreeSurfer (DK and DKT protocols), BrainSuite, and BrainGyrusMapping. DK: Desikan-Killiany; DKT: Desikan-Killiany-Tourville; BGM: BrainGyrusMapping.*

***
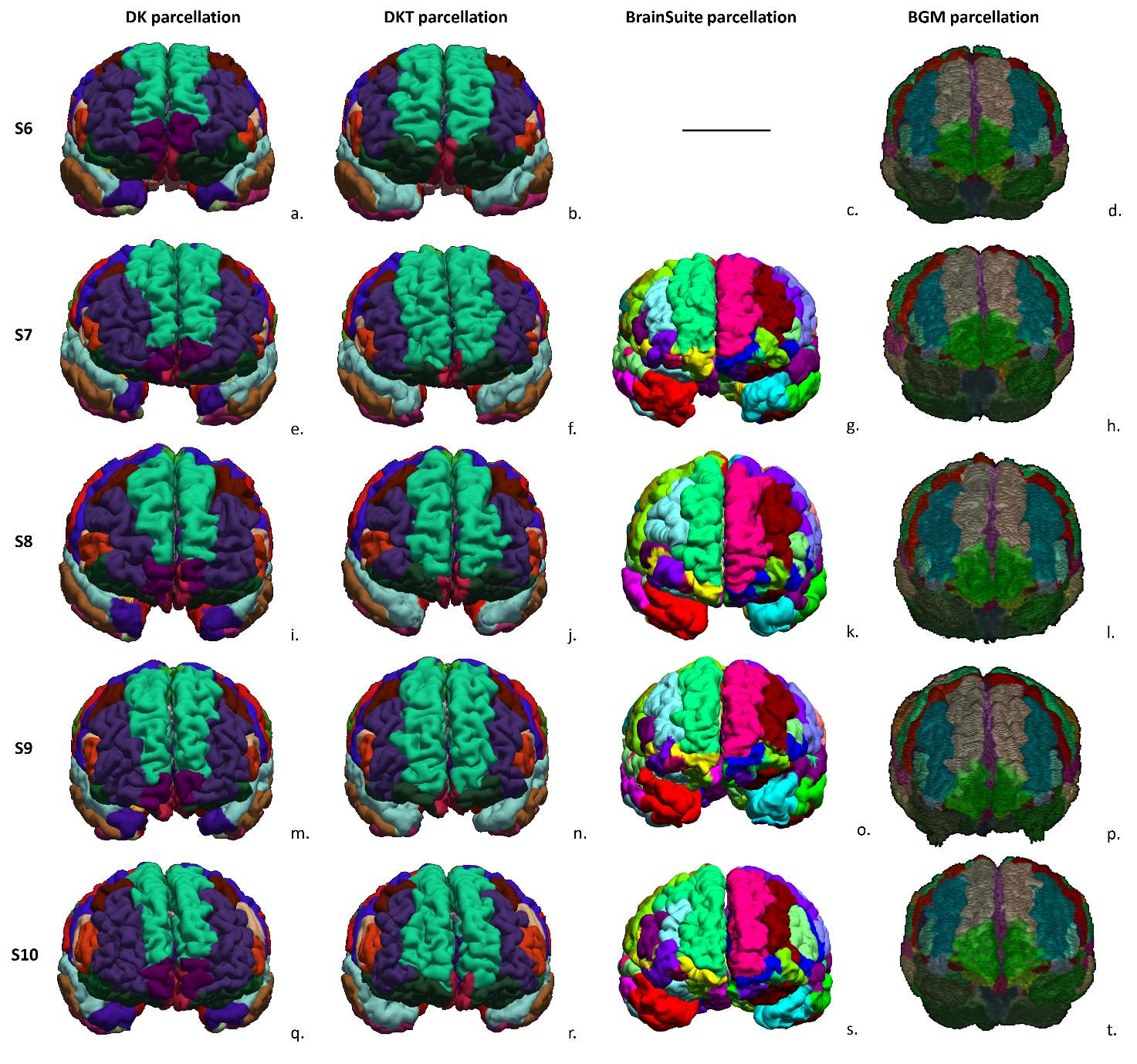
Figure S12.*** *Antero-superior view of cortical parcellation for subjects 6-10. Parcellation was done according to FreeSurfer (DK and DKT protocols), BrainSuite, and BrainGyrusMapping. DK: Desikan-Killiany; DKT: Desikan-Killiany-Tourville; BGM: BrainGyrusMapping.*
